# The effects of in-hospital orthogeriatric care on health-related quality of life: a systematic review and meta-analysis

**DOI:** 10.1093/ageing/afaf106

**Published:** 2025-04-20

**Authors:** Karin Vleeshouwers, Jole Beert, Annelies Boonen, Daisy J A Janssen, Marian Dejaeger, Mieke Deschodt, Maaike L De Roo, Bart Spaetgens

**Affiliations:** Department of Internal Medicine, Division of General Internal Medicine, Section Geriatric Medicine, Maastricht University Medical Centre, Maastricht, The Netherlands; Department of Geriatrics, University Hospital Leuven, Leuven, Belgium; Department of Rheumatology, Maastricht University Medical Centre, Maastricht, The Netherlands; Public Health Research Institute (CAPHRI), Maastricht University, Maastricht, The Netherlands; Department of Health Services Research and Department of Family Medicine Care and Public Health Research Institute (CAPHRI), Maastricht University, Maastricht, The Netherlands; Department of Research and Development, Ciro, Horn, The Netherlands; Department of Geriatrics, University Hospital Leuven, Leuven, Belgium; Department of Public Health and Primary Care, KU Leuven, Leuven, Belgium; Department of Public Health and Primary Care, KU Leuven, Leuven, Belgium; Competence Centre of Nursing, University Hospital Leuven, Leuven, Belgium; Department of Geriatrics, University Hospital Leuven, Leuven, Belgium; Department of Public Health and Primary Care, KU Leuven, Leuven, Belgium; Department of Internal Medicine, Division of General Internal Medicine, Section Geriatric Medicine, Maastricht University Medical Centre, Maastricht, The Netherlands

**Keywords:** orthogeriatric care, hip fractures, health-related quality of life, frail older people, systematic review, older people

## Abstract

**Background:**

Orthogeriatric care has been shown to effectively reduce mortality and morbidity and has a potential impact on health-related quality of life (HRQoL). This systematic review and meta-analysis summarises the effects of orthogeriatric care on HRQoL in hip fracture patients.

**Methods:**

The review protocol was registered in International Prospective Register of Systematic Reviews (PROSPERO): CRD42021206280. We searched Medline and EMBASE from inception to January 2024 without language restrictions. We included randomised and non-randomised controlled trials comparing HRQoL in older hip fracture patients receiving orthogeriatric care to other fracture care. Study quality was evaluated using the Revised Cochrane Risk-of-Bias (RoB) tool or the Newcastle–Ottawa Scale (NOS). Pooled standardised mean differences (SMDs) were calculated using random-effects models. We reported according to the Preferred Reporting Items of Systematic reviews and Meta-Analyses guidelines.

**Results:**

Eight studies involving 2411 patients were included, all employing various orthogeriatric care models with moderate to good methodological quality, based on the RoB tool and NOS. However, substantial clinical heterogeneity was present due to variations in study design, number and execution of intervention components, outcome measures and patient populations. Despite this variability, meta-analysis showed that in-hospital orthogeriatric care, compared to usual care, led to a small but statistically significant improvement in overall HRQoL (SMD 0.18, 95% CI 0.06–0.30) with moderate heterogeneity (*I*^2^ = 47%).

**Conclusion:**

In-hospital orthogeriatric care has a small but significant effect on HRQoL. This study highlights the need for clear descriptions of orthogeriatric care models, their implementation, fidelity and contextual factors. High-quality future research is essential to advance clinical practice, refine care models, address methodological limitations and prioritise patient-centred short- and long-term HRQoL outcomes.

## Key Points

- This is the first systematic review and meta-analysis to evaluate the effects of in-hospital orthogeriatric care on health-related quality of life (HRQoL).
- Orthogeriatric care has a small yet statistically significant positive impact on HRQoL.
- High-quality research is essential for improving clinical practice and refining care models
- Future research must address methodological limitations and prioritise short- and long-term HRQoL outcomes

## Introduction

With a rapidly ageing global population, we face the challenge of managing a growing number of frail older people [1]. A significant issue in this context is the increased risk of falls leading to fractures, associated with considerable mortality, morbidity and substantial healthcare costs [2]. Orthogeriatric care integrates geriatric and surgical expertise to improve patient outcomes, particularly those with hip fractures [3]. This multidisciplinary approach between orthopaedic surgeons, geriatricians and allied health professionals can be delivered through various care models. Moderate-quality evidence suggests that orthogeriatric care reduces the length of hospitalisation, in-hospital and 1-year mortality and delirium, with potential complication and cost reduction, though its impact on functional outcomes remains unclear [4].

Despite these promising findings, most research in orthogeriatric care has focused primarily on objective metrics such as mortality, morbidity and length of hospital stay [4–6]. These clinical endpoints often do not correspond with the outcomes that are most relevant to the patients' perceptions of their quality of life or goals of care [7]. In essence, (frail) older patients frequently place a higher priority on the quality rather than the duration of life [8]. This issue is exemplified by the findings of the FRAIL-HIP study, which indicated that non-operative management—although linked with higher mortality—might still be appropriate for certain patients [9]. This underscores the need for a more patient-centred evaluation of orthogeriatric care, including health-related quality of life (HRQoL). HRQoL is a patient-reported outcome that refers to the impact of an individual’s health on their ability to perform daily activities and enjoy life, covering physical, mental and social aspects [10]. Events such as fractures, pre-existing health conditions, post-operative complications and subsequent physical impairments can significantly affect independence and HRQoL [11]. Gaining insight into the effect of treatment strategies on HRQoL and the factors influencing these outcomes will encourage a more patient-centred approach, improve evaluations of treatment efficacy, including cost-effectiveness, and is essential for shared decision-making [12]. Therefore, this systematic review and meta-analysis aims to evaluate the impact of in-hospital orthogeriatric care vs usual care on HRQoL in this frail population.

## Methods

### Design

We conducted a systematic review and meta-analysis, which was registered in the International Prospective Register of Systematic Reviews (PROSPERO): CRD42021206280 [13]. We reported according to the Preferred Reporting Items of Systematic reviews and Meta-Analyses (PRISMA) guideline [14].

### Search strategy

A systematic search of the literature was performed without language restriction in Medline via PUBMED and EMBASE from inception until January 2024. The search strategy focused on the core search terms ‘frailty’, ‘fractures’, ‘co-management’ and ‘quality of life’ using MeSH terms and free text words (Supplementary Table S1). Reference lists of all articles included for full-text retrieval and of relevant systematic reviews were cross-referenced to retrieve additional relevant papers. Citation search was performed for all included articles.

### Selection of relevant papers

All studies targeting patients without specific age restrictions receiving orthogeriatric care in a hospital setting were considered. There were no restrictions regarding whether surgery was performed or not. Inclusion required a clearly specified collaboration between at least an orthopaedic surgeon and a geriatrician in a hospital setting. All types of orthogeriatric care models were considered: (i) an orthopaedic surgeon-led model in an orthopaedic ward with systematic geriatric consultations, (ii) a geriatrician-led model in a geriatric ward with regular orthopaedic input and (iii) a co-management model, where both teams share decision-making and responsibility equally. Furthermore, a validated measurement instrument specifically designed to evaluate HRQoL had to be used. We included all intervention studies, both randomised and non-randomised, with a control group (usual care or historical cohort).

### Study selection

Two reviewers independently evaluated the titles and abstracts of retrieved articles for full-text review and removed duplicates using Rayyan.ai [15]. Then, they independently assessed the full texts of studies that appeared potentially relevant, documenting reasons for exclusion for each paper. When there was no consensus, two senior reviewers were consulted. Any disagreements were deliberated by the review team until consensus was reached. Reasons for study exclusion are detailed in Supplementary Figure S1.

### Data extraction

Data extraction was performed by one reviewer and checked by a second reviewer. A third reviewer was consulted for resolution if needed. The extracted study characteristics were as follows: first author, year of publication, country, study design, study period, study population (number of patients, mean age and sex distribution of both the intervention and control groups), a detailed description of the orthogeriatric care model used and data on the predetermined outcomes, including follow-up durations.

### Quality assessment

**Figure 1:**
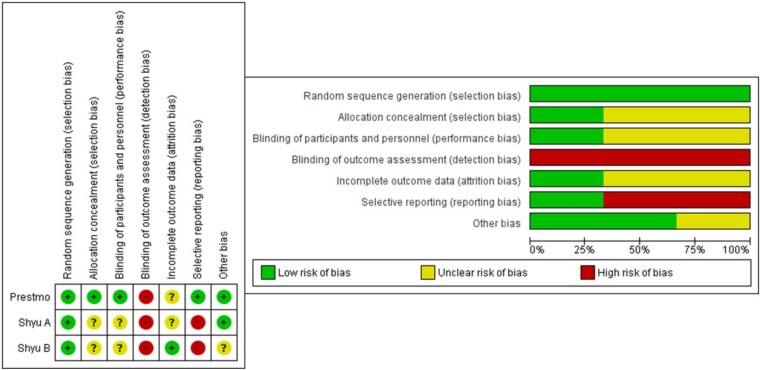
Quality assessment of the randomised studies.

Assessment of the methodological quality of the included studies was performed by two authors independently. The Revised Cochrane Risk-of-Bias tool for randomised trials (RoB 1) was applied to randomised trials, while the Newcastle–Ottawa Scale (NOS) was used for non-randomised studies [16, 17]. The NOS scores range from 0 to 9, with scores of ≥7 indicating high quality, 5–6 moderate quality and ≤4 low quality. Consensus on quality scores was achieved through discussion. The results of the quality assessments are summarised in Figure 1 (randomised controlled trials) and Table 1 (non-randomised studies).

**Table 1:** Quality assessment non-randomised studies.

	Selection				Comparability	Outcome			Total score
Publication, year (country)	Representativeness of exposed cohort	Selection of non-exposed cohort	Ascertainment of exposure	Demonstration—outcome not present at start	Controls and adjusted	Assessment of outcome	Was follow-up long enough	Adequacy of follow-up of cohorts	
Blauth et al, 2021 (Multicentre: Austria, Spain, USA, The Netherlands, Thailand, Singapore)		x	x		x	x	x		**5**
Flikweert et al, 2021 (The Netherlands)	x	x	x		x	x	x		**6**
Gao et al, 2023 (China)	x	x	x		x	x	x	x	**7**
Lizaur-Utrilla et al., 2014 (Spain)	x	x	x		x	x	x	x	**7**
Kalmet et al., 2019 (The Netherlands)	x	x	x		x		x		**5**

### Data synthesis

The extracted data were summarised into three tables for clarity. Table 2 displays the study characteristics for each included study. Table 3 provides details on the orthogeriatric care models used and lists the specific components and timing of these interventions (preoperative, postoperative and post-discharge). Supplementary Table S2 summarises the outcomes of individual studies along with their respective time points.

**Table 2:** Characteristics of the included studies.

**First author (year)**	**Country**	**Study design**	**Study period**	**Number of patients**			**Age (years + SD or IQR, as reported)**		**% Male**		**Inclusion–exclusion criteria**
				Total	Intervention	Control	Intervention	Control	Intervention	Control	
Blauth (2021)	Austria, Spain, the USA, the Netherlands, Thailand and Singapore	Prospective cohort study	2015–17	281	142	139	81.9 (SD 6.6)	83.9 (SD 6.9)	29.6%	23%	≥70 years with operatively treated proximal femur fracture
Flikweert (2021)	The Netherlands	Prospective controlled trial	2012–13	357	188	169	78 (SD 10)	80 (SD 9)	39%	31%	≥60 years, multi-trauma excluded
Gao (2023)	China	*Post hoc* analysis of prospective cohort study	2018–19	223	125	98	92 (IQR 91–94)	92 (IQR 90–93.25)	39.8%	27.2%	≥90 years with hip fracture (that completed follow-up)
Prestmo (2015)	Norway	RCT	2008–10	397	198	199	83.4 (SD 5.4)	83.2 (SD 6.4)	27%	26%	>70 years, home-dwelling, with hip fractures able to walk 10 m before fracture
Shyu A (2005, 2010)	Taiwan	RCT	2001–04	162	80	82	77.4 (SD 8.2)	78.9 (SD 7.3)	31%	32%	≥60 years, receiving hip arthroplasty or internal fixation, able to perform against gravity, pre-fracture Barthel Index >70
Shyu B (2013)	Taiwan	RCT	2005–10	299^a^	CC: 99	UC: 99 SC: 101	Total population 76.2–76.9 SD or subdivision intervention vs control not reported		Total population 32.7%–40.4% Subdivision intervention vs control not reported		≥60 years, receiving hip arthroplasty or internal fixation, able to perform against gravity and pre-fracture Barthel Index >70
Lizaur-Utrilla (2014)	Spain	Prospective cohort study with matched historical group	2011	294	153	141	82.5 (SD 7.4)	82.5 (SD 7.9)	31%	26%	≥65 years, exclusion criteria were another trauma, ASA grade 5 and contraindication to anaesthetic or surgical treatment.
Kalmet (2019)	The Netherlands	Retrospective cohort study	2012 and 2015	398	182	216	83.4 (SD 7.4)	82.2 (SD 7.5)	29%	29%	≥65 years, with surgically treated low-energy hip fracture

^a^Study with three arms.

Abbreviations. CC, comprehensive care. SC, subacute care as intervention. UC, usual care.

**Table 3:** Description and components of the orthogeriatric care models.

**Study**	**Intervention** ^**a**^	**Control** ^**a**^	**Description of the orthogeriatric care model**	**Domains of intervention**								**Timing of intervention**		
				Time to surgery	Medical	Fall & fracture assessment	Functional (physiotherapy)	Nursing care	Nutrition	Mental health/cognition	Discharge planning	Preoperative	Postoperative	Post-discharge
Blauth (2021)	Geriatric fracture centre	Usual care Centre	Interdisciplinary care or ‘geriatric fracture centres’ (GFC). GFCs had predefined treatment paths for older trauma patients, providing a fast track in the emergency department, facilitating daily communication among specialists, ensuring regular preoperative and postoperative visits by a geriatrician, and supporting daily physiotherapy and access to social workers.	x	x		x				x	x	x	
Flikweert (2021)	Comprehensive multidisciplinary care pathway	Usual care	The comprehensive care pathway includes multidisciplinary collaboration among trauma surgeons, orthopaedic surgeons, anaesthesiologists and geriatricians. It features a shared preoperative workup protocol and perioperative anaesthesiological risk assessment. Surgery is scheduled at 8 a.m. the day after admission, allowing patients to eat until midnight, with postoperative care coordinated with physical therapists and nursing home physicians, and a follow-up at a specialised outpatient clinic 6 months post-surgery.	x	x		x	x	x	x	x	x	x	x
Gao (2023)	Orthogeriatric co-management	Traditional consultation	Upon admission, patients entered an orthogeriatric ward co-managed by orthopaedic and geriatric doctors, focusing on early operation (<48 h), comorbidity evaluation and management, secondary fracture prevention, pressure sore prevention, physical therapy and early discharge. Geriatricians handled preoperative evaluation, comorbidity management, prevention of postoperative complications and secondary fracture prevention, while orthopaedic surgeons prepared and executed the surgery.	x	x	x	x	x	x		x	x	x	
Prestmo (2015)	Comprehensive geriatric care	Orthopaedic care	The clinical pathway emphasises a systematic and interdisciplinary approach before and after surgery, including comprehensive medical assessment and treatment. It focuses on somatic health (comorbidity management, drug regimen review, pain, nutrition, etc.), mental health and function, with early initiation of rehabilitation and discharge planning. The comprehensive geriatric care unit also maintained a higher staff-to-bed ratio compared to the orthopaedic care unit.	x	x	x	x	x	x	x	x	x	x	
Shyu A (2005, 2010)	Interdisciplinary intervention programme	Usual care	This programme included geriatric consultation services, a continuous rehabilitation programme and discharge-planning services. Geriatric consultations aimed to detect medical and functional problems, reduce surgery delays and provide recommendations on surgery timing and postoperative management. The rehabilitation programme offered early postoperative rehabilitation and at-home exercises, while discharge planning ensured the continuity of care with necessary referrals and follow-up monitoring.	x	x	x	x	x	x	x	x	x	x	x
Shyu B (2013)	Interdisciplinary comprehensive care programme	Usual care	Builds upon the previously mentioned model (Shyu A). Integrates the components of the subacute care model with additional health-maintenance interventions, including systematic fall-risk assessments, nutritional consultations and depression screening and management.	x	x	x	x	x	x	x	x	x	x	x
Lizaur-Utrilla (2014)	Comanagement	Usual care	The shared acute care programme for hip fracture patients involves preoperative and postoperative care managed by orthopaedic surgeons and a team of internal medicine specialists. This includes educating healthcare professionals, ensuring rapid administrative transitions and providing daily assistance, along with discharge planning. Medical intervention focuses on stabilising comorbidities, preventing and treating complications, and providing standardised nursing care and physiotherapy.		x		x	x			x	x	x	
Kalmet (2019)	Multidisciplinary clinical pathway	Usual care	The multidisciplinary clinical pathway encompasses patient care from the emergency department to discharge to a rehabilitation unit or nursing home, involving an orthopaedic trauma surgeon, a geriatrician, an anaesthesiologist and a physiotherapist. The team aims to perform surgical treatment within 24 h of admission and achieve discharge within 4 days, with agreements for transferring patients to rehabilitation facilities. The postoperative protocol emphasises early mobilisation and full weight bearing.	x	x		x				x	x	x	

^a^As described in the article.

### Meta-analysis

A meta-analysis was conducted to quantify the impact of in-hospital orthogeriatric care, compared to usual care, on HRQoL. HRQoL outcomes were categorised into physical, mental and combined subgroups, as the 36-item Short-Form Health Survey (SF-36) and its 12-item version (SF-12) generate distinct physical component summary (PCS) and mental component summary (MCS) scores [18, 19]. Pooled data were analysed using standardised mean differences (SMDs). When studies did not report mean and standard deviation, study authors were contacted. A random-effects model was applied a priori, regardless of heterogeneity (*I*^2^), to account for baseline differences, diverse outcome measures and variability in usual care [20]. A sensitivity analysis was conducted, restricting follow-up to ≤6 months. All analyses were performed using Review Manager 5.3.

## Results

### Search results

The database search identified 966 records. After removing 266 duplicates, 670 titles with abstracts were evaluated. Full-text examination of the remaining 20 articles resulted in the inclusion of nine articles. We included one additional article after the reference search in Supplementary Figure S1.

### Study characteristics

The ten included papers reported findings from eight unique studies (Table 2) [21–30]. Three papers reported data analysis from the same data set originally collected by Shyu et al. and reported results from the pilot study and a 2-year follow-up, with the latter divided into two separate articles [25–27]. These separate publications are considered collectively as a single study in the subsequent results section, involving up to 162 unique patients. Of the eight unique studies, three were randomised controlled trials [24–28], and five were non-randomised studies [21–23, 29, 30], of which three had a prospective [21, 22, 29] and two a retrospective design [23, 30].

In total, these studies included 2411 unique patients; among them, 1268 (52.6%) received orthogeriatric care. The mean age was 82.0 years, with 81.7 years for those receiving orthogeriatric care and 82.4 years for the usual care group. The majority of the patients were women (69.4%; 67.7% in orthogeriatric care and 71.3% in usual care). The mean duration of follow-up across the studies was 14 months, ranging from 6 months to 2 years. All studies focused on hip fracture patients, and several applied additional inclusion and exclusion criteria. For example, studies were restricted to patients who—prior to the fracture—could walk more than 10 m [24], had a Barthel Index above 70 [25–28] or were over 90 years old [23]. Some studies ruled out patients with multiple traumas [22]. Prestmo et al. excluded nursing home residents and patients with pathological fractures, multiple traumas or a short life expectancy [24].

### Methodological quality

The methodological quality of the randomised studies was assessed as moderate to good (Figure 1). Unclear risks of bias were primarily due to the challenges of blinding in studies that measure HRQoL as outcome. Consequently, this issue did not significantly influence our overall assessment, which concluded that the randomised studies had moderate to good quality. The non-randomised studies similarly received moderate to good quality ratings, with median scores of 6 and ranging from 5 to 8 points (Table 1).

### Description of the Orthogeriatric care models

Table 3 provides an overview of the orthogeriatric care models. Although the descriptions are relatively broad and general, occasionally to the extent that clear categorization within the three predefined models from the methods is not feasible, the key components can still be extracted. However, the specific implementation of these components was often unclear. Notably, these components consistently highlight several key similarities. In each of the studies, a geriatrician is prominently involved in the intervention group, with active participation noted both before and after surgery. All models comprehensively incorporate physiotherapy, either through daily sessions or through a standardised regimen. Notably, six of the studies highlight the importance of initiating early discharge planning and rehabilitation services soon after surgery. Fast-track services in the emergency department or the provision of early surgery within 24 to 48 h of admission are emphasised in four studies, with one study specifically allocating a designated time slot for these surgeries. Nutritional consultation or management is identified as a crucial component in five of the studies. Measures for secondary fracture prevention and fall-risk assessment are addressed in three and four studies, respectively. Comorbidity management features in five studies, with two of these studies particularly focusing on distinguishing between high-risk and low-risk patients following a consultation by a geriatric nurse. Medication review is mentioned in only one study. Additionally, three studies include mental health screening or management as part of their care model.

### Health-related quality of life—instruments

Of the eight studies, four used the EuroQol-5 dimensions (EQ-5D) with or without the EQ-Visual Analogue Scale (EQ-VAS), two used the SF-36 and two used the SF-12 [18, 19, 31]. The EQ-5D assesses five dimensions: mobility, self-care, usual activities, pain/discomfort and anxiety/depression, with scores ranging from no problems to extreme problems. The EQ-VAS rates current health on a 0–100 scale [31]. The SF-36 and SF-12 cover eight domains: physical functioning, role limitations due to physical health, bodily pain, general health perceptions, vitality, social functioning, role limitations due to emotional problems and mental health, which are categorised into PCS and MCS [18, 19, 31]. Notably, not all studies reported raw HRQoL data, as detailed in Supplementary Table S2, which presents the findings of the individual studies. Furthermore, in the study by Lizaur-Utrilla et al., it was unclear how the historical HRQoL data were obtained, complicating the interpretation of their findings [29].

### Effect of orthogeriatric care on HRQoL—findings from individual studies

First, two studies identified HRQoL as their primary outcome [28, 30], while the other six studies considered it a secondary outcome. Notably, Shyu et al. were the only authors to report performing a formal power calculation, but only for the physical functioning scale of SF-36 [28]. For the remaining studies, it remained unclear whether they were adequately powered to evaluate HRQoL as an outcome measure. Second, the studies yielded mixed results regarding the impact of orthogeriatric care on HRQoL. Three studies demonstrated a statistically significant positive effect of orthogeriatric care on HRQoL, while the others showed no significant impact. The studies that found a significant benefit in HRQoL, favouring the intervention group, were the three RCTs. A first RCT by Prestmo et al. reported higher EQ-5D-3L scores at 4 and 12 months for the comprehensive geriatric care group (0.54 and 0.52) compared to the usual care group (0.46 and 0.45) [24]. Similarly, RCTs by Shyu et al. found significant improvements across various SF-36 domains over time for the comprehensive care group [25–28]. Additionally, the PCS score was higher in the comprehensive care group. Further, comprehensive care improved PCS score at 3 months (beta 2.22, *P* < .05) and 12 months (beta 4.1, *P* < .05). The follow-up at 24 months indicated a 6.08 point score on PCS health in the intervention group (*P* < .001).

Regarding orthogeriatric care models and their specific components, it is notable that the three studies demonstrating a positive effect also incorporated the most specific components (Table 3). These included a targeted focus on comorbidities, pre- and postoperative visits by the geriatric team, daily physical therapy, nutritional assessments, early initiation of rehabilitation and early discharge planning. Additionally, these models addressed mental health, including depression screening and management. However, no significant effects on MCS were found. Furthermore, it is worth noting that other studies implementing a similar number of components and the (almost) same orthogeriatric care elements did not report a statistically significant positive impact on HRQoL.

### Meta-analysis

**Figure 2:**
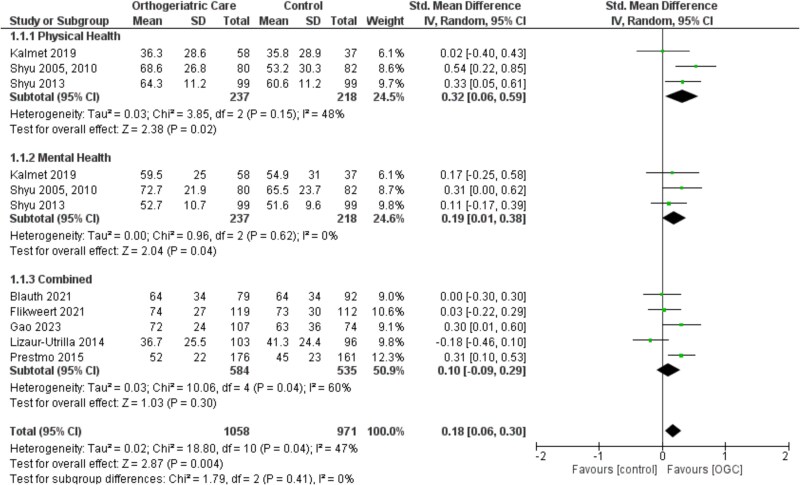
Meta-analysis of in-hospital orthogeriatric care on HRQoL compared to usual care. Physical health refers to the PCS score of the SF-36 or SF-12. Mental health represents the MCS score of the SF-36 or SF-12. The combined category is based on the EQ-5D, which does not differentiate between physical and mental health. Additionally, one study (Lizaur-Utrilla 2014) reported a total SF-12 score. Abbreviation. OGC, orthogeriatric care.

All eight studies were included in the meta-analysis after additional data were provided by the authors. In-hospital orthogeriatric care, compared to usual care, was associated with a small but statistically significant improvement in overall HRQoL (SMD 0.18, 95% CI 0.06–0.30). Subgroup analyses demonstrated varying effects across physical (SMD 0.32, 95% CI 0.06–0.59; *n* = 3 studies), mental (SMD 0.19, 95% CI 0.01–0.38; *n* = 3) and combined HRQoL outcomes (SMD 0.10, 95% CI −0.09–0.29; *n* = 5). Moderate heterogeneity was observed in the overall analysis (*I*^2^ = 47%) (Figure 2).

A sensitivity analysis restricted to studies with a follow-up of ≤6 months included six studies (excluding two with follow-ups at 1 and 2 years). This analysis indicated a slight increase in the effect size (SMD 0.25, 95% CI 0.09–0.41), suggesting a somewhat greater short-term impact of orthogeriatric care on HRQoL. However, substantial heterogeneity was present (*I*^2^ = 64%), indicating variability in short-term outcomes across the studies (Supplementary Figure S2).

## Discussion

This systematic review and meta-analysis examined the impact of in-hospital orthogeriatric care on HRQoL in frail older hip fracture patients. The meta-analysis demonstrated a small statistically significant improvement in overall HRQoL compared to usual care, with the strongest effects observed in physical HRQoL. However, substantial statistical and clinical heterogeneity was present, reflecting variations in study design, intervention components and patient populations. While our findings suggest that orthogeriatric care has the potential to improve HRQoL, they also highlight persistent knowledge gaps that require further investigation.

Older adults recovering from hip fractures undergo a complex trajectory of care, where both hospital-based interventions and post-discharge support influence long-term outcomes [32]. Although our review primarily focused on in-hospital care, HRQoL several months post-surgery is likely shaped by factors such as formal and informal support, rehabilitation services and long-term care. However, these were rarely reported in the included studies, making it difficult to isolate the direct impact of orthogeriatric care from broader post-hospital influences. This challenge is compounded by limited descriptions of in-hospital interventions, creating uncertainty about their implementation and consistency across settings.

### Key methodological challenges

Several critical issues were identified from our systematic review. First, the eight included studies demonstrated considerable variability in study design, follow-up duration, in- and exclusion criteria, HRQoL instruments and timing of outcome measurement. Second, while some key similarities existed in orthogeriatric care models, detailed descriptions of their implementation and execution were frequently lacking. Third, studies with positive HRQoL effects often implemented more structured and extensive orthogeriatric care models. However, even studies with comparable interventions and moderate to good methodological quality did not consistently show HRQoL improvements. This discrepancy raises questions about the factors driving variability in outcomes, including potential differences in patient characteristics, post-discharge pathways and integration of orthogeriatric care into common practice.

### Variability in orthogeriatric care components and execution

One key explanation for these differences is the variability in care components and their execution across studies. While this review attempted to evaluate the number and type of care components, heterogeneity in their implementation, execution and reporting hindered a comprehensive understanding of their impact.

For example, orthogeriatric care was broadly defined as formal collaboration between orthopaedic surgeons and geriatricians within a hospital setting. However, this definition does not fully capture the complexity and variability of potential care components. Factors such as care setting, the duration and intensity of follow-up by geriatric specialists and the integration of rehabilitation services may influence patient outcomes. Evidence suggests that models incorporating a broader array of components generally yield better patient outcomes, supporting the idea that a comprehensive, coordinated approach can enhance HRQoL [33]. However, identifying and assessing the presence and impact of specific core components remains challenging. While some studies clearly described elements such as early discharge planning, early rehabilitation or a focus on comorbidity and medication management, their absence in other studies does not necessarily imply they were not addressed. It is possible that these aspects were managed as part of standard geriatric assessments but were not explicitly reported.

Organisational factors may also contribute to outcome variability. Specialised departments with improved staff-to-patient ratios—including dedicated geriatric nurses and physiotherapists—could enhance care quality and positively impact HRQoL. Indirect evidence from Prestmo et al. suggests that an improved staff-to-patient ratio in the intervention group was associated with better HRQoL outcomes [24]. However, other studies did not report such organisational aspects.

These findings emphasise the need for more detailed reporting on orthogeriatric care components, their implementation and fidelity in future studies to better understand their impact on HRQoL outcomes.

### Long-term impact of orthogeriatric care on HRQoL

Another challenge is determining whether the effects of in-hospital orthogeriatric care persist over time. Several studies measured HRQoL only at long-term follow-up time points, one or even two years after the intervention. This raises questions about whether in-hospital interventions can realistically continue to influence HRQoL beyond this extended period. Sensitivity analysis showed a modest increase in effect size when restricting analysis to studies with a shorter follow-up, suggesting that orthogeriatric care has a more pronounced impact in the early post-fracture phase. However, these benefits may diminish over time without sustained post-discharge interventions. This aligns with broader findings that HRQoL is shaped by multiple factors beyond hospital-based care, reinforcing that in-hospital orthogeriatric care is just one part of a larger effort to optimise long-term HRQoL.

### Baseline differences and generalisability

Baseline differences between study groups in the non-randomised studies further complicate interpretation. One particularly relevant baseline difference was fracture type, which varied between populations in three of the five non-randomised studies [22, 23, 30]. Although HRQoL outcomes were reported as similar, the lack of adjustment for fracture type may have masked potential effects. This is particularly relevant given indirect evidence suggesting that fracture type may influence HRQoL outcomes through various pathways [34].

Additionally, study inclusion and exclusion criteria challenge the generalisability of HRQoL findings to real-world populations. Some studies excluded patients unable to walk 10 m, those with a Barthel Index below 70 or nursing home residents, significantly limiting the applicability of these findings to the broader geriatric trauma population [24–28]. Future research should ensure that study populations more accurately reflect real-world clinical scenarios.

### Expanding the scope of HRQoL assessment in orthogeriatric care

Despite the increasing literature on orthogeriatric care, only eight unique studies were included in this review, highlighting a persistent focus on traditional clinical outcomes over HRQoL. The integration of patient-reported outcome measures reflects a welcome shift towards patient-centred care, offering valuable insights into intervention effectiveness and guiding treatment strategies to better align with patients’ needs and preferences. Additionally, patient-reported outcome measures enhance communication between patients and healthcare providers, facilitate shared decision-making and improve overall satisfaction with care.

However, concerns remain about whether generic instruments—such as the EQ-5D and SF-36—fully capture HRQoL in this specific patient population [35]. Instruments such as ICECAP—which includes dimensions such as independence and social relationships—may be particularly relevant for older adults recovering from fractures [36]. Therefore, future research should explore the inclusion of additional tools or compare generic and disease-specific instruments to gain a more comprehensive understanding of patient needs and the full impact of orthogeriatric interventions on HRQoL [37].

### Strengths and limitations

This was the first systematic review and meta-analysis prioritising HRQoL as an outcome in frail older hip fracture patients. A comprehensive search strategy without language restrictions and cross-referencing of included studies strengthened its validity. However, substantial statistical and clinical heterogeneity in study design, time periods, orthogeriatric care models and HRQoL assessment limited comparability. Furthermore, generalisability may be limited by the inclusion of studies from predominantly high-income countries.

### Conclusion and future directions

This systematic review and meta-analysis highlights two key insights into the impact of orthogeriatric care on HRQoL in frail older hip fracture patients. First, despite evidence suggesting a small but statistically significant improvement in HRQoL with orthogeriatric care, substantial gaps remain, emphasising the need for more high-quality research focused on HRQoL outcomes. This is particularly relevant for underrepresented groups, such as nursing home residents, for whom HRQoL may be a more meaningful outcome than traditional clinical outcomes. Second, addressing these knowledge gaps requires not only more studies prioritising HRQoL but also clearer descriptions of interventions and their components. This includes defining orthogeriatric care models, identifying effective components and conducting detailed process evaluations to understand how specific factors contribute to HRQoL outcomes. Moreover, establishing a core set of standardised outcome measures is crucial for consistency while considering healthcare system structures, post-discharge support and socioeconomic influences that shape HRQoL.

As healthcare continues to move towards more patient-centred models, outcome measures must align with the values and preferences of frail older adults. Expanding HRQoL assessments beyond generic tools to include dimensions such as independence and social relationships could provide deeper patient insights. Continued research, methodological advancements and a commitment to patient-centred care are essential for refining orthogeriatric interventions and ensuring meaningful HRQoL improvements in this vulnerable population. By prioritising patient-centred outcomes, healthcare providers can deliver more effective and impactful care for older adults.

## Supplementary Material

aa-24-2897-File004_afaf106

## Data Availability

This review is based on previously published studies, so all data used are publicly available. Relevant study data are included in the article or provided as supplementary information.
