# Supplementary material for: The effects of in-hospital orthogeriatric care on health-related quality of life: a systematic review and meta-analysis
Source: Age Ageing. 2025 Apr 20;54(4):afaf106. doi: 10.1093/ageing/afaf106 (PMC12009544; doi:10.1093/ageing/afaf106)
Supplement: aa-24-2897-File004_afaf106 [file aa-24-2897-file004_afaf106.pdf]

The effects of in-hospital orthogeriatric care on health-related quality of life: a systematic review and meta-analysis

Table of Contents

Supplementary Table 1.	Search strategy
Supplementary Figure 1.	PRISMA flowchart of study selection process
Supplementary Table 2.	Health-related quality of life outcomes
Supplementary Figure 2.	Meta-analysis of the impact of in-hospital orthogeriatric care on health-related quality of life compared to usual care at follow-up ≤6 months

Supplementary Table 1. Search strategy

PUBMED N=491

**Patient**	(“Frail Elderly”[Mesh] OR “Frailty”[Mesh] OR frail*[tiab] OR vulnerab*[tiab] OR elder*[tiab] OR older[tiab]) AND (“Fractures, Bone”[Mesh] OR “Arthroplasty, Replacement”[Mesh] OR fracture*[tiab] OR “orthopedic surgery”[tiab] OR “total hip replace*”[tiab] OR “fragility fracture*”[tiab] OR “geriatric trauma”[tiab])
**Intervention**	("Geriatric Assessment"[Mesh] OR “Geriatrics”[Mesh] OR “Geriatricians”[Mesh] OR Geron*[tiab] OR geria*[tiab] OR orthoger*[tiab] OR comanage*[tiab] OR co-manage*[tiab] OR “perioperative manag*”[tiab] OR multidisciplinary[tiab] OR interdisciplinary[tiab] OR “team approach”[tiab] OR “integrated care”[tiab] OR "fracture liaison service"[tiab])
**Control**	*Control groups involved comparisons with usual care (with or without randomization) or historical comparisons, this was not specified in the search string.*
**Outcome**	(“Quality of Life”[Mesh] OR “quality of life” OR “quality measure*” OR “health-related quality of life” OR HRQOL OR EQ-5D)

All PICO-components were combined with AND. This withheld 491 results.

EMBASE N=475

**Patient**	(‘frail elderly’/exp OR Frailty/exp OR (frail* OR vulnerab* OR elder* OR older):ti,ab,kw) AND (Fracture/exp OR ‘Replacement arthroplasty’/exp OR ‘orthopedic surgery’/exp OR ‘fragility fracture’/exp OR ‘total hip replacement’/exp OR (fracture* OR ‘geriatric trauma’):ti,ab,kw)
**Intervention**	(geriatric assessment/exp OR geriatrics/exp OR geriatrician/exp OR (geron* OR geria* OR orthoger* OR comanage* OR co-manage* OR "perioperative manag*" OR multidisciplinary OR interdisciplinary OR "team approach" OR "integrated care" OR "fracture liaison service"):ti,ab,kw)
**Control**	*Control groups involved comparisons with usual care (with or without randomization) or historical comparisons, this was not specified in the search string.*
**Outcome**	(Quality of Life/exp OR ("quality of life" OR "quality measure*" OR "health-related quality of life" OR "HRQOL" OR EQ-5D):ti,ab,kw)

All PICO-components were combined with AND, and conference abstracts were excluded (using NOT [conference abstract]/lim). This withheld 475 results.

Supplementary Figure 1. PRISMA flowchart of study selection process

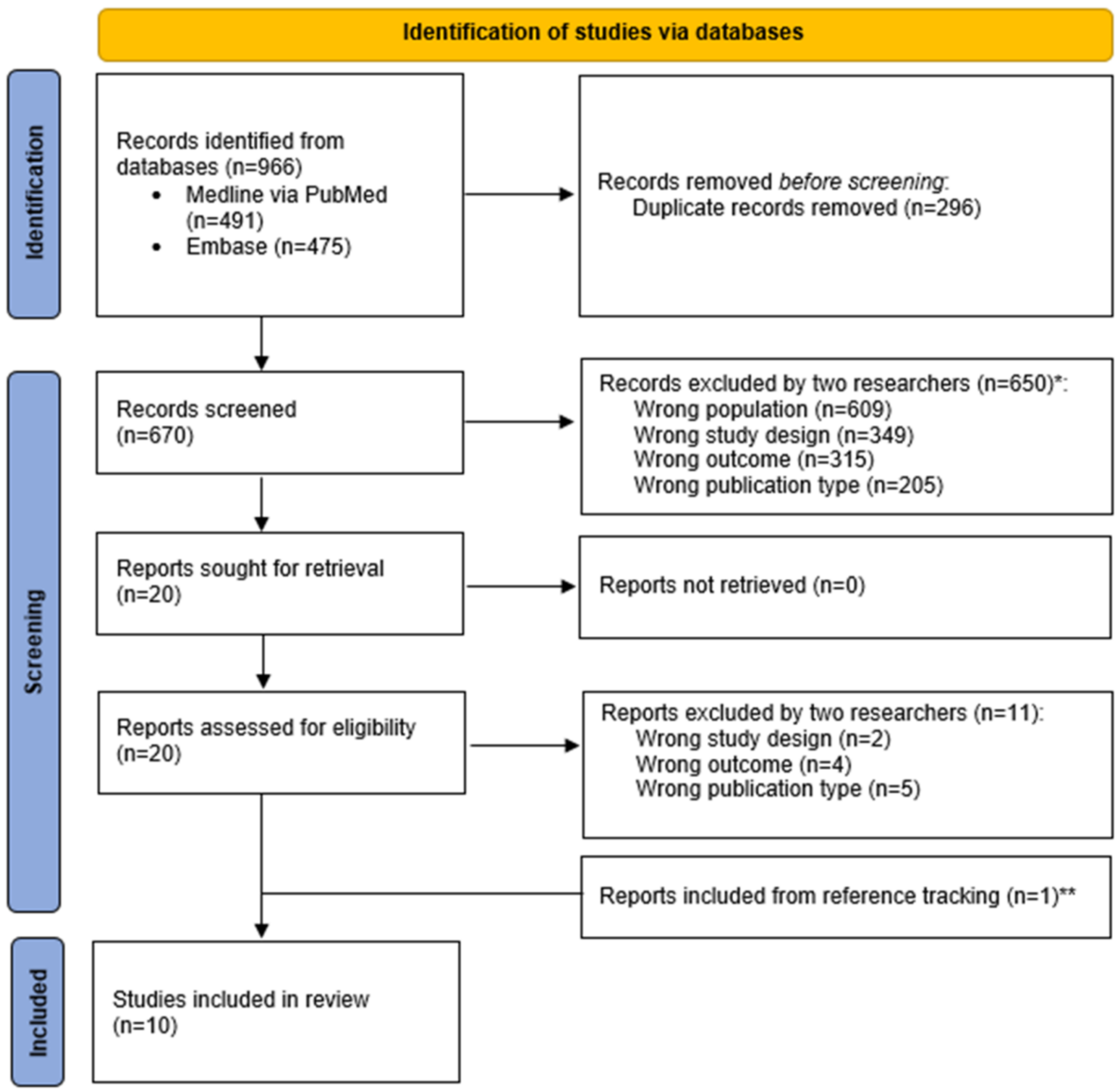

From: Page MJ, McKenzie JE, Bossuyt PM, Boutron I, Hoffmann TC, Mulrow CD, et al. The PRISMA 2020 statement: an updated guideline for reporting systematic reviews. BMJ 2021;372:n71. doi: 10.1136/bmj.n71

* The number of reasons for exclusion exceed the number of screened records because multiple reasons could be selected for exclusion

****** Reference lists of all articles included for full-text retrieval, were cross-referenced to retrieve additional relevant papers. Citation search was performed for all included articles. Reference lists of systematic reviews relevant to the field of orthogeriatric co-management were checked for additional relevant articles.

Supplementary Table 2. Health-related quality of life outcomes

**First author (year)**	**HRQoL Instruments**	**Follow-up time points**	**Conclusions**
Blauth (2021)	EQ-5D, EQ-VAS	12 weeks<br>1year	No significant difference in EQ-5D or EQ-VAS between the intervention vs. control groups at any time point
Flikweert (2021)	EQ-5D-3L	Admission<br>6 weeks<br>6 months	No significant difference in EQ-5D-3L between the intervention vs. control groups at any time point
Gao (2023)	EQ-5D-5L<br>EQ-VAS	30 days<br>1 year	Orthogeriatric co-management was linked to improved EQ-5D-5L scores after 30 days (0.53 vs 0.36, p<0.001), but this improvement was not observed at 1-year. Additionally, there were no significant differences in EQ-VAS scores between the intervention and control groups at any time point.
Prestmo (2015)	EQ-5D-3L	1 month<br>4 months<br>12 months	The EQ-5D-3L scores were higher in the comprehensive geriatric care group compared to the orthopaedic care group at both 4 months (0.54 vs 0.46, p=0.033) and 12 months (0.52 vs 0.45, p=0.015).
Shyu A (2005, 2010)	SF-36 represented in 8 domains* and summarized in PCS and MCS	1 month<br>3 months<br>6 months<br>12 months<br>18 months<br>24 months	The intervention group exhibited higher HRQoL scores in BP (75.3 vs 64.1, p=0.03), VT (67.5 vs 53.9, p<0.001), MH (67.8 vs 58.6, p=0.02), PF (48.4 vs 28.2, p<0.001), and RP (50.4 vs 28.6, p=0.006) at 3 months. After adjusting for time using a GEE model, comprehensive care showed significant improvements across five domains of the SF-36 up to 12 months: PF, RP, BP, VT, and MH.<br>Furthermore, subjects in the intervention group showed a significantly better PCS (beta=6.08, p<0.001) after 24 months in adjusted linear regressions, while MCS was not affected by the intervention.
Shyu B (2013)	SF-36 represented in 8 domains* and summarized in PCS and MCS	1 month<br>3 months<br>6 months<br>12 months	*Comprehensive care* demonstrated superior PCS scores compared to usual care at 3 months (beta= 2.22, p <0.05) and 12 months (beta= 4.10, p<0.05) in adjusted linear regression.
Lizaur-Urtrilla (2014)	SF-12, 'total score'	Pre-fracture<br>1 year	No significant differences in HRQoL between the intervention vs. control groups at any time point
Kalmet (2019)	SF-12, PCS and MCS	at least 2 years follow-up	No significant differences in HRQoL between the intervention vs. control groups at any time point

Abbreviations: PF= physical functioning, RP= role limitations due to physical health problems, BP= bodily pain, GH= general health, VT= vitality, SF=social functioning, RE= role limitations due to emotional problems, MH= mental health, PCS= Physical Component Summary, MCS= Mental Component Summary

Supplementary Figure 2. Meta-analysis of the impact of in-hospital orthogeriatric care on health-related quality of life compared to usual care at follow-up ≤6 months

	Orthogeriatric Care			Control				Std. Mean Difference
**Study or Subgroup**	**Mean**	**SD**	**Total**	**Mean**	**SD**	**Total**	**Weight**	**IV, Random, 95% CI**
**2.1.1 Physical Health**								
Shyu 2005, 2010	58.2	30.1	80	42.7	28.2	82	11.3%	0.53 [0.22, 0.84]
Shyu 2013	53.4	9.4	99	51.7	9.1	99	12.4%	0.18 [-0.10, 0.46]
**Subtotal (95% CI)**			**179**			**181**	**23.7%**	**0.35 [0.01, 0.69]**
Heterogeneity: $Tau^2 = 0.04$; $Chi^2 = 2.61$, df = 1 (P = 0.11); $I^2 = 62\%$								
Test for overall effect: Z = 2.02 (P = 0.04)								
**2.1.2 Mental Health**								
Shyu 2005, 2010	69.6	26.1	80	62.6	24.3	82	11.4%	0.28 [-0.03, 0.59]
Shyu 2013	55.1	9.1	99	55.4	8.7	99	12.4%	-0.03 [-0.31, 0.25]
**Subtotal (95% CI)**			**179**			**181**	**23.8%**	**0.11 [-0.19, 0.42]**
Heterogeneity: $Tau^2 = 0.03$; $Chi^2 = 2.13$, df = 1 (P = 0.14); $I^2 = 53\%$								
Test for overall effect: Z = 0.74 (P = 0.46)								
**2.1.3 Combined**								
Blauth 2021	59	36	96	56	33	101	12.4%	0.09 [-0.19, 0.37]
Flikweert 2021	74	27	119	73	30	112	13.1%	0.03 [-0.22, 0.29]
Gao 2023	53	24	122	36	28	88	12.3%	0.66 [0.38, 0.94]
Prestmo 2015	54	26	177	46	26	170	14.7%	0.31 [0.10, 0.52]
**Subtotal (95% CI)**			**514**			**471**	**52.5%**	**0.27 [0.01, 0.53]**
Heterogeneity: $Tau^2 = 0.05$; $Chi^2 = 12.25$, df = 3 (P = 0.007); $I^2 = 76\%$								
Test for overall effect: Z = 2.05 (P = 0.04)								
**Total (95% CI)**			**872**			**833**	**100.0%**	**0.25 [0.09, 0.41]**
Heterogeneity: $Tau^2 = 0.03$; $Chi^2 = 19.60$, df = 7 (P = 0.006); $I^2 = 64\%$								
Test for overall effect: Z = 3.04 (P = 0.002)								
Test for subgroup differences: $Chi^2 = 1.11$, df = 2 (P = 0.57), $I^2 = 0\%$								

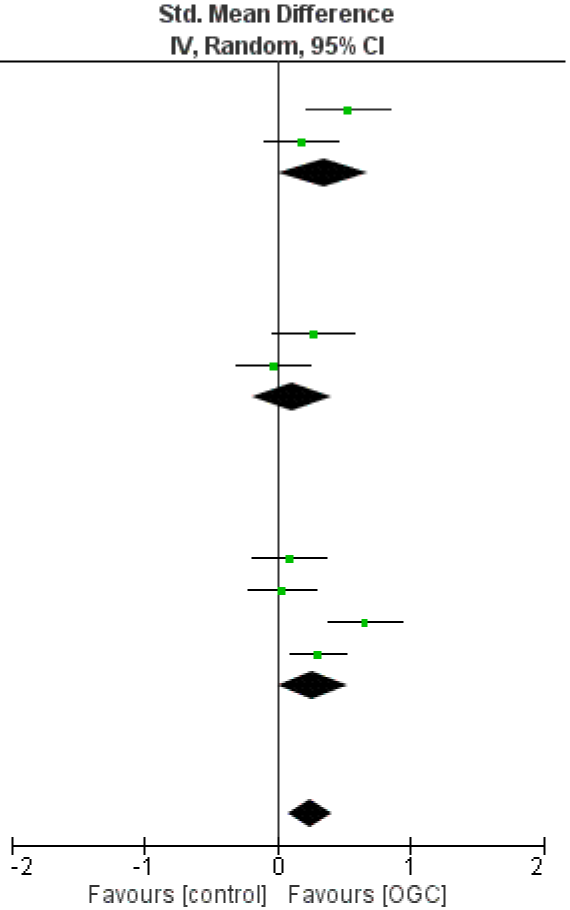

Legend: Physical Health refers to the Physical Component Summary Score of the SF-36 or SF-12. Mental Health represents the Mental Component Summary Score of the SF-36 or SF-12. The Combined category is based on the EQ-5D, which does not differentiate between physical and mental health. Additionally, one study (Lizaur-Urtrilla 2014) reported a total SF-12 score. Abbreviations: OGC = Orthogeriatric Care
